# Intestinal CD8^+^ tissue‐resident memory T cells: From generation to function

**DOI:** 10.1002/eji.202149759

**Published:** 2022-08-29

**Authors:** Liqing Cheng, Simone Becattini

**Affiliations:** ^1^ Department of Pathology and Immunology, Faculty of Medicine University of Geneva Geneva Switzerland; ^2^ Geneva Centre for Inflammation Research, Faculty of Medicine University of Geneva Geneva Switzerland

**Keywords:** anatomical niches, intestinal Trm, intestinal tumor, mucosal immunology, tissue imprint

## Abstract

Tissue‐resident memory T cells (Trm), and particularly the CD8^+^ subset, have been shown to play a pivotal role in protection against infections and tumors. Studies in animal models and human tissues have highlighted that, while a core functional program is shared by Trm at all anatomical sites, distinct tissues imprint unique features through specific molecular cues. The intestinal tissue is often the target of pathogens for local proliferation and penetration into the host systemic circulation, as well as a prominent site of tumorigenesis. Therefore, promoting the formation of Trm at this location is an appealing therapeutic option. The various segments composing the gastrointestinal tract present distinctive histological and functional characteristics, which may reflect on the imprinting of unique functional features in the respective Trm populations. What these features are, and whether they can effectively be harnessed to promote local and systemic immunity, is still under investigation. Here, we review how Trm are generated and maintained in distinct intestinal niches, analyzing the required molecular signals and the models utilized to uncover them. We also discuss evidence for a protective role of Trm against infectious agents and tumors. Finally, we integrate the knowledge obtained from animal models with that gathered from human studies.

## Graphical abstract

Tissue‐resident memory T cells are key players in protection from infectious agents and tumors at multiple anatomical locations. Here, we review the current knowledge about gut‐resident CD8^+^ T cells, discussing the ontogeny, phenotype, and functions of these cells across the entire length and distinct tissue layers of the intestinal tract.

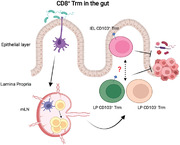

## Introduction

Tissue‐resident memory T cells (Trm) are a specialized subset of T cells that reside in nonlymphoid organs and that can rapidly reactivate following cognate antigen encounter [1]. Trm display distinct phenotypic and functional features with respect to circulating central (Tcm) and effector memory T cells (Tem) [2]. Over the past decade, numerous studies have shown the importance of Trm in combatting infectious agents and tumors. The key regulatory networks underlying generation and maintenance of Trm cells at distinct anatomical sites, including unique recruitment and specification signals, have been only partially unraveled. In this context, while common core molecular pathways for Trm regulation have emerged, Trm at distinct anatomical niches have proven to rely on tissue‐specific signals, which imprint unique functional traits [3].

The gastrointestinal (GI) tract represents one of the largest mucosal surfaces in the human body and a prominent site for pathogen invasion and tumor development. In addition, it also regulates physiology, given its role in nutrient absorption and interaction with the gut microbiota. As an important mucosal organ and a site of immune response, CD8^+^ Trm cells can also be found in the gut, where they play critical roles in immune surveillance against intestinal infections and tumors.

Here, we primarily review studies addressing the generation, maintenance, and function of CD8^+^ Trm in the GI tract, emphasizing their tissue‐specific signatures, as well as the experimental models utilized to investigate their formation.

## Discovery and early studies on Trm

The intestinal tissue is now recognized as one of the major reservoirs of Trm, with strong implication for pathogenesis of diseases, immunization approaches, and immune homeostasis [4]. The organization and cellular composition of the intestinal tissue vary across the length of the GI tract. However, a common structure can be detected, with a monolayer of columnar epithelial cells intermingled with mucus‐producing Goblet cells and cells specialized in the secretion of antimicrobial peptides. Underneath this layer is the lamina propria (LP), abundant in immune cells and placed immediately above a submucosa, which separates the mucosa from the lower muscular layers [5]. Immune cells, including Trm, can accumulate both in the epithelial layer, which contains Intra‐Epithelial Lymphocytes (IEL), and in the LP.

Much of what we currently know about intestinal Trm has been extrapolated from experiments carried out in animal models of infection, particularly systemic viral infections, although orogastric administration of bacteria has also been employed.

The formal demonstration of the existence of a tissue‐resident, non‐recirculating population of memory CD8^+^ T cells following HSV infection was published in 2009 [6], however, multiple studies had already described the migration and long‐term retention of T cells in the intestinal tissue.

Early studies from the laboratory of Leo Lefrançois employed systemic infection with VSV‐OVA to demonstrate that pathogen‐specific CD8^+^ T cells rapidly and robustly accumulate in the small intestine, particularly in its epithelial layer. These cells were assumed to be bona fide memory T cells as they could be detected in the tissue for up to 120 days, and unlike memory T cells found in the peripheral lymph nodes, they expressed the gut‐homing receptor αEβ7 and lacked CD62L [7, 8].

Shortly after, tetramer staining was used to track the local kinetics of antigen‐specific T cells in models of orogastric infection with Listeria monocytogenes (Lm) [9] or an engineered Lm strain expressing the model antigen OVA (Lm‐OVA) [10, 11]. Huleatt et al. showed that *per os* (p.o.), as compared to intravenous (i.v.) administration of Lm resulted in higher accumulation of pathogen‐specific T cells in the LP and epithelium of the small intestine (SI), but lower accumulation in the spleen [9]. Although these distinct infection routes required profoundly different infectious doses, resulting in uneven intestinal Lm burdens (higher in the p.o. group), infected mice displayed comparable systemic Lm levels across groups. In these experiments, the TCR repertoire of intestinal memory T cells generated via oral versus i.v. infection differed substantially, suggesting some level of segregation between the tissue‐resident population and the circulating T cell pool [9].

In agreement with the above data, in the first study employing Lm‐OVA as a model infectious agent, Pope et al. demonstrated that p.o. infection generates a substantial expansion of pathogen (OVA)‐specific T cells in the intestinal tissue, and suggested that these cells could rapidly reactivate, expanding locally and producing effector cytokines upon pathogen re‐challenge [10].

Two landmark papers published around the same time shed new light on the magnitude and relevance of the non‐lymphoid tissue (NLT)‐resident memory T cells. In particular, immunization with VSV or OVA adjuvanted with LPS, resulted in superior accumulation of antigen‐specific memory CD8^+^ and CD4^+^ T cells, respectively, in non‐lymphoid organs than in the spleen, with the small intestine resulting in a major reservoir in this process [11, 12]. CD8^+^ T cells recovered from the intestinal LP displayed higher cytotoxic potential than those obtained from spleen and strong IFN‐γ production capacity [11], consistent with what was shown elsewhere (9]. Importantly, high levels of accumulation of CD8^+^ T cells in NLT could be achieved both through p.o. and i.v. infection (using Lm‐OVA and VSV, respectively), suggesting that distinct pathogens and infection routes could be interchangeably employed to promote efficient generation of NLT‐migrating cells [9, 11]. Of note, although far less studied than their CD8^+^ T counterparts, gut CD4^+^ Trm cells have been also efficiently induced in infection models, with the two populations displaying both shared as well as unique phenotypic characteristics, for example, CD4^+^ and CD8^+^ Trm cells expressed high levels of CD69, while CD4^+^ expressed lower level of CD103 compared to CD8^+^ Trm cells [13].

## Migration and homing markers of intestinal T cells

In order to home to the intestine, primed T cells must express selected migratory receptors (Table 1).

**Table 1:** Markers of tissue‐resident memory T cells

**Marker**	**Up/down**	**Function**	**Site**	**Notes**	**Mouse**	**Reference**	**Human**	**Reference**
CCR9	↑	migration	SI>LI		√	[18]		
α4β7	↑	migration	SI, LI?	early upregulation, decreased in memory phase	√	[levels that from 21, 22]		
CXCR3		migration	SI? LI		√	[24]		
CD103	↑	binds to E‐cadherin	SI, LI	necessary for cell accumulation but not retention	√	[22]	√	[25]
CD69	↑	inhibits S1pr1	SI, LI	dispensable for Trm retention in mouse intestine	√	[26]	√	[25]
CD49a	↑	binds to collagen IV	skin	not studied in intestine	√	[27]	√	[28]
S1pr1	↓	cell egress	SI, LI?	regulated by KLF2	√	[29]		
S1pr5	↓	cell egress	skin	not studied in intestine	√	[30]		
CCR7	↓	LN homing	skin	not studied in intestine	√	[31]		
**Transcription** **factors**								
Hobit	↑	inhibits cell egress	SI, LI?	in humans, it can be expressed by circulating memory cells and liver resident NK cells	√	[32]	√	[33, 34]
Blimp1	↑	inhibits cell egress	SI, LI?	early upregulation, lower level in Trm	√	[32]		
Runx3	↑	promotes residency	SI, LI?		√	[35]		
Bhlhe40	↑	promotes Trm mitochondrial fitness and epigenetic programming	Lung and tumor		√	[36]		
Junb/Fosl2	↑	not well studied	SI, LI?		√	[37]		
Klf2	↓	promotes cell egress	SI, LI?		√	[29]		

A seminal study from von Andrian and colleagues established that dendritic cells (DCs) from the Peyer Patches have a greater potential to imprint gut‐homing phenotypes in T cells, by promoting the upregulation of the migratory receptors CCR9 and α4β7 [14], mostly due to the capacity of producing the dietary vitamin A‐derived metabolite retinoic acid [15]. CCR9‐ligand, the chemokine CCL25, is mostly produced by epithelial cells of the small intestine [5]; the α4β7 integrin, instead, binds to MAdCAM‐1, which is constitutively expressed on high endothelial venules in mesenteric lymph nodes, as well as on venules in the LP of SI and LI, becoming strongly up‐regulated at these locations upon inflammation [16, 17].

Interestingly, it was reported that CD11c^+^ DCs from small and large intestines imprint primed T cells with the ability to preferentially migrate to their respective anatomical site of origin. In fact, SI DCs promoted stronger expression of CCR9 on T cells, while colonic DCs would mostly induce α4β7 expression, possibly due to lower expression of Retinal dehydrogenase (RALDH), an enzyme that converts vitamin A into retinoic acid; as a result, anti‐CCR9 antibodies could inhibit migration of transferred T cells to the SI, but not to the colon [18].

In a model of Lm p.o. infection, it was further shown that migratory DCs expressing the transcription factor Batf3, but not MLN‐resident DCs, are necessary for the priming of gut‐resident memory T cells, possibly by transporting bacterial particles to the MLNs [19]. However, another report showed that stromal MLN cells, rather than dendritic cells, are responsible for imprinting of a gut‐homing phenotype in T cells [20].

Sheridan et al. established an oral infection model with Lm to monitor the endogenous CD8^+^ T cell response by specific tetramer staining in spleen, mLN, lung, intestine IEL, and LP. Interestingly, although cells activated in the spleen were previously shown to be able to migrate to the intestine [21], the authors found that performing splenectomy prior to infection had no impact on the numbers of intestinal memory Trm recovered [22]. MLNs were confirmed to be the primary CD8^+^ T cell priming site following p.o. Lm infection, and T cell migration to the intestinal LP and epithelium relied, at least in part, on CCR9 and α4β7 expression. Migration of T cells to the intestinal tissues can be promoted also by inflammatory chemokines such as CXCL9 and CXCL10, which mediate recruitment to the SI and LI in models of orogastric infection [23, 24].

The capacity of cells primed in the mLN to express α4β7 and migrate to the intestine varies over time, peaking around d4.5 in the LCMV infection model, at least in transfer experiments utilizing unstimulated mice as recipients. The expression of α4β7 is then lost overtime, regardless of the infection model used [21].

P.o. infection favored the accumulation of memory precursor effector cells (MPEC, defined as CD127^+^KLRG1^−^ [38]) in the intestinal tissue as compared to spleen, while intranasal immunization generated intestinal effector cells with short‐lived effector cells (SLEC) phenotype (CD127^−^KLRG1^+^) that were rapidly lost; the relative MPEC/SLEC ratio could not be further modulated by the infectious dose, highlighting that distinct infectious routes have a prominent role in determining the subsequent cell fate, a concept validated in other tissues for CD4^+^ T cells [22, 39].

CD103, an integrin that has long been used as a marker to identify Trm, particularly at epithelial surfaces, interacts with E‐cadherin, and has been shown to contribute to the establishment of intestinal Trm both in the small and large intestine [40, 41]. CD103 was initially reported to be necessary for retention, but not for accumulation, of CD8^+^ T cells in the SI epithelial layer [42]; however, a later report suggested that CD103 confers an initial advantage in terms of recruitment to the epithelium, but is not required for later maintenance, as co‐transfer of WT and CD103 deficient T cells upon infection produced an uneven ratio of cells lodging in the epithelium, which however remained constant at later time points [22]. While these discrepancies remain to be clarified, all the above studies demonstrated an important role for CD103 in establishing gut‐resident Trm.

CD49a (Integrin β1, or very late antigen 1 (VLA1)), a subunit of the collagen receptor α1β1, is also well‐known to favor Trm maintenance in epithelial tissues such as the skin by binding to type I and IV‐ collagen [43], and was found to be up‐regulated in human gut Trm [44] and necessary for maintenance of Trm in the SI epithelium in mice [45].

Establishment of the intestinal Trm program is a gradual process, as initially proposed by flow cytometric analyses on transferred transgenic T cells [46], and recently confirmed by overtime single‐cell (sc) RNA‐seq in SI IEL following infection [47]. This concept was first established in skin infection models which demonstrated sequential acquisition of CD69 and CD103 by skin infiltrating CD8^+^ T cells, two key molecules for retention of Trm at this location [31], but also up‐regulated by gut‐homing Trm [47].

Together with CD103, CD69 has been used as bona fide marker of tissue residency, but its relative importance in the acquisition of a Trm phenotype is still debated. For instance, in mice CD69 was found to be completely dispensable for the establishment of Trm in the intestine as well as at many other anatomical locations, while it was strongly needed for lodging of Trm into the kidneys [26]. On the contrary, human studies have suggested that CD69 is a reliable marker for gut residency in T cells [48].

Persistence of Trm in the tissue relies not only on the expression of molecules such as those mentioned above, but also on the selective down‐regulation of specific receptors, most notably the sphingosine‐1‐phosphate receptor (S1PR1), whose expression is driven by the transcription factor KLF2, and that prompts cells to rejoin the circulation on the basis of S1P chemotactic gradient [29]. Of note, CD69 interferes with S1PR1 and inhibits its function, which has long been considered its major contribution to establishing Trm [49, 50]. Similarly, CCR7, which is a lymphoid organ‐homing chemokine receptor responding to the CCL19/20 gradient, is also down‐regulated upon activation of Trm transcriptional programs, as part of a common core signature shared by Trm across tissues, including the gut [44, 47, 51].

Once they have seeded the intestinal tissue, CD8^+^ Trm become rather limited in their capacity to move, as elegantly shown in experiments employing photoconvertible Kaede mice [52]. In particular, while intestinal Trm display some degree of freedom of movement in the local tissue, they do not appear to migrate from small intestine to large intestine, or vice versa; on the contrary, bi‐directional migration of Trm was observed between the LP and the epithelial layer within given intestinal segments [52].

It is important to note that, although most intestinal Trm do not appear to recirculate at steady state largely as a result of the above transcriptional program [13, 21], two recent studies showed that, upon pathogen reinfection, Trm can proliferate locally and generate a progeny of Tem cells that return to the circulation, substantially contributing to protection [53, 54]. Thus, the fate of Trm is partly reversible and Trm maintain a certain degree of plasticity. On the contrary, upon reinfection, memory CD8^+^ Tcm appear to be scarcely capable of seeding the intestinal tissue and generating Trm [55].

## Signals that drive intestinal Trm generation and long‐term retention

**Figure 1:**
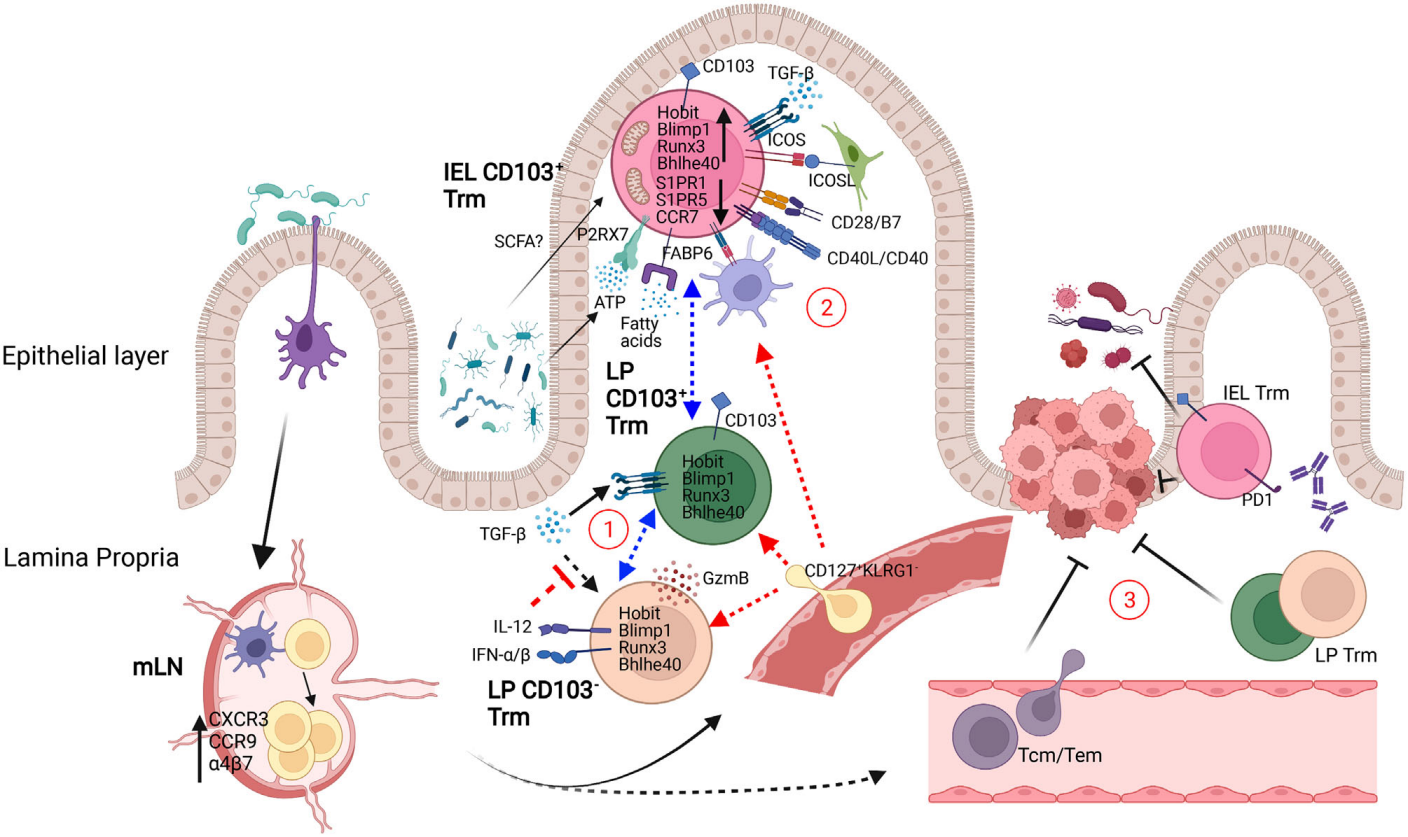
**Schematic representation of the generation of intestinal CD8+ Trm**. Upon intestinal infection, dendritic cells (DC) capture pathogen particles or fragments and migrate to the MLN, where they present antigens to naïve CD8+ T cells. Activated T cells upregulate integrins and chemokine receptors, such as CCR9, α4β7, and CXCR3, which allow them to migrate to the inflamed intestine and differentiate into Trm cells upon entering the tissue. This process involves activation of a specific transcriptional program which is acquired in a gradual manner, and likely relies on tissue‐specific cues. Such cues produce the emergence of distinct subsets of intestinal CD8+ Trm, characterized by unique phenotypic (such as expression of CD103) or functional (such as cytokine and cytotoxic molecule production) features. Further, these cells occupy distinct niches within the tissue. We identify three outstanding questions that remain to be addressed in the field and are depicted with circled numbers in the figure. (1) What is the detailed developmental pathway for LP and IEL Trm in the different intestinal segments? (2) Is local antigen encounter required to establish a residency program in the intestine? (3) What is the protective potential of gut Trm against infectious agents and local tumors, with respect to that of circulating T cells, and can gut‐resident Trm be harnessed through immune checkpoint blockade? *(Created with BioRender.com)*

A variety of intrinsic and extrinsic signals can contribute to instructing the intestinal Trm fate (Figure 1). Here, we review those signals that to date have been shown to play an important role for Trm formation and maintenance.

### Specifying transcription factors

In a landmark study, Mackay and collaborators identified Hobit and Blimp1 as the key transcription factors regulating maintenance of Trm cells within the skin by promoting cooperative repression of tissue egress‐associated genes, such as the lymph node homing receptor CCR7, and the transcription factor KLF2, which regulates S1PR1 (Table 1) [32]. Consistently, in a LCMV infection model, Hobit and Blimp1 deficiency caused diminished Trm accumulation in the small intestine LP and epithelium, which was already apparent ten days and became highly significant fifty days post‐infection. These transcription factors showed an additive effect, possibly because they regulate largely overlapping sets of genes, although with distinct kinetics, and double knock‐out mice displayed a more pronounced Trm deficiency than individual knock‐out [56, 57].

Subsequent research focused on exploring the unique transcriptional regulating network involved in Trm formation, including additional transcription factors. In addition to Hobit and Blimp1, Runx3 was also reported to be critical in regulating T cell residency in the intestine, and its overexpression promoted T cell infiltration into the tumor microenvironment and control of tumor growth (Figure 1) [35].

Two recent works employed scRNA‐seq to investigate the overtime expression of specifying genes in SI IEL in LCMV‐infected mice, and uncovered a previously unappreciated level of heterogeneity in intestinal Trm [47, 58]. Kurd and colleagues identified transcription factors with no previously reported role in Trm cells such as *Nr4a2*, *Junb*, and *Fosl2*, whose knockdown impaired Trm formation. The authors described that as early as 4 days post‐infection, T cells lodging into the epithelial layer of the SI display unique transcriptional features that are coherent with a Trm program, and evolve further in the following weeks. The confirmed signature included genes such as CD69, CD103, CCR9, and CXCR3, but also P2RX7, Fabp1‐2‐6 (see below), and genes involved in the TCR signaling pathway (*Zap70*, *Itk*, *Lats2)*, regulators of intracellular calcium (*Orai1*, *Orai2*, *Sri*, and *Rrad*), and regulators of NFAT and NF‐κB signaling [47]. At these early stages, the authors could identify a subset of Trm precursors characterized by low expression of IL2R‐α (IL‐2Rα^lo^), which showed an enhanced capacity to generate Trm. Later on, two major subsets of SI IEL Trm could be detected, based on the relative expression of CD28, with CD28^hi^ cells also expressing higher levels of CD127 as compared to CD28^lo^ cells [47].

Other transcription factors such as *Ikzf2*, *Ikzf3*, *Gata3*, *Irf4*, and *Id2* were also upregulated during Trm development in this study, but their relative contribution to gut Trm formation remains to be investigated [47].

Similarly, Milner et al. unraveled the heterogeneity of the SI IEL population generated following infection, and identified at least two distinct subsets of Trm, which could be detected already at seven days post‐infection, and showed well‐distinct profiles [58]. The first subset, defined as Blimp1^hi^Id3^lo^, comprised cells with a canonical effector phenotype, characterized by the expression of *Cx3cr1, Zeb2, Klrg1, Id2*, and production of inflammatory cytokines and granzymes; the second, defined as Blimp1^lo^Id3^hi^, presented features of memory‐precursor cells, such as expression of *Bcl6*, *Bach2*, *Tcf7*, and *Cd127*. As predicted by their phenotype, Blimp1^lo^Id3^hi^ cells displayed greater potential to generate new Trm cells in transfer experiment. Interestingly, this study found that Id3 deletion alone did not impact the generation of such memory subset, likely due to the redundant activity of Id2. Accordingly, combined Id2 and Id3 deficiency, caused severe loss of all memory cells, particularly the CD127^hi^ subset [58].

The stress‐responsive transcription factor Bhlhe40 was also found to contribute to Trm maintenance via metabolic regulation and generation of substrates for epigenetic modifications. Bhlhe40 can regulate the expression of Runx3, Notch1, and Notch2, which are necessary for Trm cell formation and residency, as well as the expression of mitochondria‐associated genes [36].

### Extrinsic signals

Multiple local signals of different nature contribute to the specification and maintenance of intestinal Trm (Figure 1).

#### Costimulatory molecules

Several costimulatory molecules have been implicated in the generation of Trm populations in the intestine. CD40‐CD40L interaction and signals from CD4^+^ T cells were recognized as crucial for intestinal memory CD8^+^ T cell generation, as Lm‐OVA p.o. immunization of CD40^−/−^ or MHCII^−/−^ mice resulted in dramatic halting of OVA‐specific T cell accumulation in the intestinal lamina propria and epithelium, but not in the spleen [10]. Along similar lines, while the costimulatory molecule B7‐1 was fundamental to produce memory T cells in both LN and intestinal tissue, B7‐2 was found to play a key role only in the latter one [7].

Upon LCMV infection, the co‐stimulatory receptor ICOS is highly expressed by intestinal CD8^+^ Trm [59], in keeping with reports identifying this molecule as a signature of Trm [31]. Notably, ICOS was required for resident but not circulating CD8^+^ memory T cell generation in this systemic infection model, by impacting the initial establishment of tissue Trm. Bone marrow chimera experiments proved that ICOSL expression in radioresistant cells is responsible for its effect. Mechanistically, ICOS‐mediated downregulation of KLF2 (which regulates S1PR1 expression) and PI3K signaling were shown to contribute to Trm establishment, although through incompletely elucidated pathways [59].

#### Cytokines

A crucial role for TGF‐β in tissue retention of Trm has been shown in multiple tissues, including skin and gut [31, 51, 60]. In fact, TGF‐β signaling induces CD103 expression and promotes Trm maintenance across organs [43]. Zhang et al. reported two distinct functions for TGF‐β signaling during the formation and maintenance of intestinal Trm in a model of systemic infection with the LCMV strains Armstrong (Arm) and Clone 13, which promote acute and chronic infection, respectively. In acute infection, TGF‐βRII deficient cells accumulated less efficiently than WT T cells in the epithelium and LP of the small intestine, and were completely lost over time. On the contrary, in chronic infection, WT and TGF‐βIIR deficient cells equilibrated to comparable numbers. Absence of TGF‐β signaling impaired expression of retention markers, such as CD103, CD69, integrin β7, all of which are strongly induced by the cytokine. However, lack of TGF‐β signaling resulted in enhanced expression of the integrin α4β7 at early time points in Clone 13‐, but not Arm‐infected mice, leading to increased migration to the gut in the former group, which at least partially accounted for the differential effect observed in the two infection models. This effect relied on the sustained migration of cells from the LN in the chronic model because virus clearance, which occurred months later, resulted in disappearance of TGF‐βRII KO cells from the gut of Clone13‐infected mice as well [61]. The importance of TGF‐βR signaling for the formation of Trm in the small intestine LP and epithelium was confirmed in a model of oral infection with Lm‐OVA, where it was shown to be crucial not only with respect to CD103 and CD69 expression, but also to the early generation of MPEC seeding the intestinal tissue [22].

A strong signature of TGF‐βRII signaling activation was also detected in a recent scRNA sequencing study, as early as four days post LCMV infection, in SI IEL CD8^+^ as compared to activated splenic T cells [47].

While TGF‐β is extremely important, not all intestinal Trm populations seem to depend upon it. Bergsbaken et al. found that oral infection with *Yersinia pseudotuberculosis* induces a population of CD69^+^CD103^−^ CD8^+^ T cells in the LP of the small intestine, but not in the IEL, which is TGF‐β‐independent, as deficiency of TGF‐β receptor in T cells did not affect CD103^−^ Trm cells generation in the LP [23]. These cells form clusters with CD4^+^ T cells and CX3CR1^+^ mononuclear phagocytes around areas of bacterial infection. This process of localization is dependent on CXCL10/CXCR3 signaling, and CXCR3 deficiency hampered antigen‐specific T cell accumulation in the SI LP by selectively impairing the development of the CD103^−^ population [62].

In a subsequent study aimed at dissecting the specific signals modulating intestinal Trm generation in this model, it was shown that the inflammatory cytokines IFN‐β and IL‐12, which are induced during intestinal infection, counter TGF‐β‐induction of CD103 expression in effector T cells. IL‐12 was produced by newly recruited CCR2^+^ monocytes. *In vivo*, IL‐12R and IFNAR‐deficient T cells could be primed and proliferated normally but failed to differentiate into CD69^+^CD103^−^ cells, and IL12R KO cells were not retained in the tissue [63].

Of note, unequal responsiveness to TGF‐β was recently confirmed to be a major driver of Trm diversity, with respect to CD103 expression and functional properties, in multiple organs besides the intestine [3].

IL‐15, a cytokine with well‐studied effects on memory CD8^+^ T cells, was instead shown to be dispensable for the maintenance of CD8^+^ Trm in the intestine as well as in other tissues [64].

#### Metabolic factors

Uptake and utilization of specific metabolites are important to establish memory T cells, whose metabolism is known to be distinct from that of naïve and effector cells, with a higher utilization of fatty acid oxidation and oxidative phosphorylation as opposed to aerobic glycolysis [65]. In this regard, Trm represent no exception, as beautifully exemplified by the study of Pan et al. who employed VACV‐OVA virus skin infection to show that the fatty‐acid‐binding proteins FABP4 and FABP5, transcriptionally regulated by PPAR‐γ, are highly upregulated in skin Trm as compared to Tcm or Tem cells [66]. Deficiency in these two receptors decreased fatty acid uptake and impaired Trm long‐term maintenance and survival, by hampering fatty acid oxidation [66]. Expanding on these findings, Frizzell et al. found that different isoforms of fatty acid‐binding proteins in Trm cells are induced at different anatomical locations. For example, FABP1, FABP2, and FABP6 were highly expressed in the SI IEL, with Fabp2 and Fabp6 being uniquely expressed at this location [67], and would be up‐regulated by transferred formerly liver‐resident Trm upon migration to this tissue.

In a following study, Li et al. reported the transcription factor Bhlhe40 to be critical in Trm metabolic regulation. In an influenza lung infection model, Bhlhe40 deficiency did not affect effector CD8^+^ T cell generation in the early phase however, generation of Trm, unlike circulating memory cells, was greatly compromised in *Bhlhe40^−/−^* mice. The function of Trm cell, as indicated by IFN‐γ production, was also diminished in knockout animals. RNA‐seq analyses revealed that Bhlhe40 regulated the expression of genes associated with mitochondrial membrane and metabolism, such as mitochondria complexes‐related genes. OXPHOS was downregulated, while mitochondrial damage was increased in Bhlhe40‐deficient cells. The defect in mitochondrial function led to the decreased production of metabolites associated with cell fueling and epigenetic modification, in particular the decrease of acetyl‐Co A, which can affect histone acetylation and expression of effector molecules [36].

P2RX7‐mediated signaling, which is activated by sensing of extracellular ATP, is also required for the generation and maintenance of long‐lived memory CD8^+^ T cells via pathways that include, but are not limited to, metabolic rewiring. P2RX7 deficiency led to decreased mitochondrial mass in T cells in a mouse model, as well as to lower oxygen consumption rates. In addition, P2RX7 can also control the intestinal CD8^+^ Trm sensitivity to TGF‐β, as CD103 expression was decreased in the absence of P2RX7 following LCMV infection [68, 69].

#### Gut microbiota

An important player in intestinal physiology and immunity, the gut microbiota can impact Trm generation, although the breadth of its effects and underlying mechanisms are still being actively investigated [70].

Providing a strong proof of principle for microbiota influence on Trm, Honda and collaborators showed that reconstitution of mice with a rationally‐designed consortium of commensal bacteria, promoted expansion of systemic and local CD8^+^ T cell populations, including gut Trm in the small intestine and colonic LP [71]. This effect was mediated by bacterial activation of a variety of immune pathways, including IFNGR signaling, as well as antigen presentation by CD103^+^ DCs. Reconstitution of mice with the CD8^+^ T cell‐enhancing bacterial consortium resulted in heightened protection of the intestinal tissue from oral Lm infection, suggesting that augmentation of gut Trm protective functions had been achieved, although a contribution of circulating cells could not be excluded in these experiments [71].

Bachem et al. revealed that microbiota‐derived short‐chain fatty acid, such as butyrate, promote the transition of activated CD8^+^ T cells to memory by enhancing fatty acid uptake and oxidation, thus regulating mitochondrial function and cellular metabolism. However, while this mechanism was shown to impact splenic memory T cells, its relative contribution to gut Trm formation was not investigated [72].

Another Trm‐modulating signal provided, at least in the intestine, by bacteria is ATP, which is sensed by T cells through the purinergic receptor P2RX7 [73, 74]. Henrique et al. reported P2RX7 directs metabolic fitness of CD8^+^ T cells, promoting the generation of long‐lived central memory cells [69]. Notably, upon LCMV infection, P2RX7 was later found to be highly expressed in Trm as compared to Tcm and Tem cells. Mechanistically, ATP sensing via P2RX7 enhanced T cell sensitivity to TGF‐β, which induces CD103 expression and promotes residency [68]. Antibiotic treatment lowered the intestinal concentration of ATP affecting survival of specific T cell subsets, such as Tfh and CD8^+^ Trm [68, 73, 74], although in the case of the latter population, microbiota depletion produced only a partial decrease, indicating that alternative sources of this molecule must exist in the gut.

Despite the above evidence suggesting the importance of a healthy gut microbiota to achieve strong CD8^+^ Trm responses, this notion may not apply to all scenarios.

We previously reported that temporary disruption of microbiota‐mediated colonization resistance via treatment with streptomycin or other antibiotics, favors the intestinal expansion of orally‐administered antigen‐engineered Lm, enhancing the generation of antigen‐specific CD8^+^ Trm in the small and large intestine, a strategy we named TMDI (Transient Microbiota Depletion‐boosted Immunization) [24, 75]. This model could be used to dissect the molecular requirements for Trm generation and accumulation in the intestinal tissue, including LP and epithelium of the large intestine, which has received far less attention than the SI.

Interestingly, and consistent with our data, Cho et al. recently showed that p.o. infection with Lm‐OVA resulted in much higher intestinal expansion of transferred OTI cells in germ‐free mice than in SPF mice, further questioning the importance of microbiota signals to promote intestinal Trm generation [76]. In these experiments, Lm was retained in the intestine of gnotobiotic animals *ad libitum* without causing tissue damage, due to the adaptive downregulation of virulence factors occurring in the bacterium, which was in turn driven by the presence of antigen‐specific T cells [76]. This is particularly interesting considering that repeated administration of streptomycin (an antibiotic to which the laboratory Lm strain 10403s is resistant) boosted Trm production up to 100‐fold as compared to standard oral infection [24], likely by delaying the recovery of the microbiota and thus sustaining antigen presentation, collectively suggesting that gut‐adapted strains of Lm could be used as extremely efficient mucosal vaccine platforms.

In this context, it is worthwhile noting that antibiotics have been shown to promote immunization efficacy and Trm accumulation even through microbiota‐independent mechanisms. Gopinath and collaborators elegantly showed that topical administration of some, but not all aminoglycosides, promoted clearance of viruses in the female genital tract (HSV‐2) and lungs (IAV), while oral administration did not produce such effect. Neomycin, but not streptomycin or amikacin, which have a different chemical structure, could activate an IFNAR‐independent but TLR3‐, TRIM‐, and IRF3/7‐dependent pathway leading to the recruitment of dendritic cells to the site of infection. Mechanistically, neomycin might make self RNA more prone to be recognized by TLR3 [77]. This effect was preserved in GF animals, demonstrating a lack of influence of the microbiota. In a later publication, topical application of neomycin in the vagina of mice was also shown to promote the recruitment of virus‐specific CD8^+^ T cells primed via subcutaneous injection, similarly to what previously shown for CXCL10 [78, 79]. Neomycin ‘pull’ increased accumulation of CD69^+^CD103^+^ local Trm and enhanced protection from subsequent infections to a similar extent as CXCL10. Thus, the selection of alternative antibiotics in TMDI might potentially enhance even further the recruitment of T cells to the intestine by coupling microbiota‐dependent and ‐independent mechanisms.

#### Antigen dose and tissue localization

The importance of local antigen presentation in the establishment of CD8^+^ Trm is debated, and very little work has been carried out in this regar in the intestinal tissue.

In skin, in vitro activated gBT‐I T cells transferred into naïve mice, were recruited and differentiated into Trm in the flank of the animal treated with the inflammatory molecule 2,4‐dinitrofluorobenzene (DNFB), but not in the untreated flank, leading to the conclusion that Trm can be established in the absence of specific local antigen [80]. However, a side‐by‐side comparison to establish the relative efficiency of recruitment and persistence of T cells in the presence of antigen, was not performed in this study.

Similarly, in a model of transfer into Rag‐deficient mice, OTI cells were shown to persist in the intestine for at least a month in the absence of antigenic stimulation, although ‘emptiness‐driven’ expansion may not accurately recapitulate the scenario encountered by authentic Trm cells in the tissue [42].

Contrary to what suggested above, two elegant studies made a strong case for the importance of local antigen presentation in the establishment of Trm [81, 82]. By adopting combined i.v. and local (ear) infection with VACV expressing model antigens, it was shown that while initial recruitment of virus‐specific CD8^+^ T cells relies uniquely on inflammation and is independent on antigen, only T cells that are exposed again to the antigen in the local environment, upon migration to the tissue, would proceed forming memory and being retained. Antigen recognition in the tissue correlated with increased expression of CD69 and promoted competition amongst T cells with distinct specificities when viral antigens were presented by the same APC. These results uncovered a mechanism by which APC may regulate the occupation of specific tissue niches by T cells displaying the highest chances of recognizing antigens expressed in that particular tissue [81, 82].

With respect to the intestinal tissue, and in keeping with the above publications, our work suggested that antigen load is a particularly critical factor in the establishment of large CD8^+^ Trm pool. By leveraging an oral infection model in which the strength of inflammation was maintained constant, while the amount of provided antigen varied over two orders of magnitude, we could show a direct correlation between antigen dose and Trm accumulation in the intestinal tissue [24]. However, it remains to be established whether such excess of antigen acts in the tissue favoring re‐stimulation of Trm precursors upon migration to the intestine, or it rather impacts priming in the MLNs.

Notably, Svensson and collaborators reported that, in an in vitro priming system, high antigen doses decreased the ability of intestinal DCs to promote up‐regulation of CCR9 and α4β7 in T cells, impairing their homing to the intestinal tissue upon transfer into mice [83]. One possible explanation to reconcile these findings with our data, is that in the model we adopted, the CXCL9‐10/CXCR3 axis plays a strong role in the recruitment of T cells to the intestinal tissue, as indicated by reduced Trm accumulation in CXCR3 KO mice, making CCR9 and α4β7 potentially less important for gut homing [24].

## Functional differences in Trm across intestinal sections and layers

Early studies utilizing c‐Kit‐ or SCF (c‐Kit ligand)‐deficient mice demonstrated a differential impact of these molecules on T cells residing in the small and large intestine, and hinted at a distinct developmental origin and migratory pattern for IELs in these compartments, as well as between LP and IEL within each of these two districts [84]. By employing intrarectal infection with modified vaccinia virus Ankara (MVA), Isakov et al. further investigated such relationship and found that LP CD8^+^ T cells displayed the highest functional avidity, followed by those obtained from the spleen and then from the IEL, which displayed the lowest. Along similar lines, by comparing the TCR‐Vβ usage and CDR3 length, the authors detected substantial dissimilarity between the repertoire of cells accumulating in the LP and IEL, suggesting that these cells might be not only displaying distinct functional properties, but also having different clonal origin and limited cross‐talk, despite the proximal anatomical location [85]. On the contrary, another study reported clear evidence for T cell bidirectional movement between SI IEL and LP, as visualized by intravital microscopy [52]; this behavior would be expected to produce a certain degree of clonal overlap between the two layers of the intestinal tissue, in agreement to what shown in human intestine [48].

## Intestinal Trm functions: Cytokine production, cytotoxic potential, and proliferation

Trm are known to have unique functional capacities with respect to circulating T cells, and recent work comparing Trm recovered from the liver, spleen, and salivary glands has reinforced the notion that even across organs and tissues, the phenotype and functions of these cells varies greatly as a result of specific local cues [3].

A systematic and exhaustive comparison of the functional capabilities of intestinal Trm versus T cells in the circulation or those residing in different organs has never been conducted, but sparse insights can be gathered from the literature.

Several studies have concluded that T cells residing in the intestine have stronger cytotoxic potential then splenic or circulating memory T cells. This was first shown in a side‐by‐side assessment of splenocytes and T cells recovered from the SI lamina propria, despite these populations produced IFN‐γ to similar extents [86]. An identical trend was observed across multiple organs, with Trm from lung and liver also showing greater lytic capacities than splenocytes [86]. Intestinal IEL resulted high in expression of Granzyme B when compared to Trm from other compartments and to splenocytes, although they produced lower amount of cytokines such as IFN‐γ and TNF‐α with respect to the latter [42, 46, 64, 87] but similar levels to those produced bt Trm in the female reproductive tract (FRT) and salivary glands [88]. Further, GzmB levels were much higher in the SI IEL and SI LP Trm than in T cells from peripheral blood, salivary gland, and FRT [53].

Along similar lines, SI LP Trm showed higher GzmB expression levels than Trm from the female reproductive tract following LCMV infection, both in the CD8 and in the CD4 compartments [13]. Within the human intestinal tissue, CD103^−^ cells displayed the highest cytotoxic potential, assessed via perforin and GzmB staining, followed by CD103^+^ LP cells, and finally CD103^+^ IELs, consistent with other reports in the mouse [48]. CD103^+^ LP cells, on the other hand, displayed stronger IFN‐γ production potential [48].

Partly in contrast with the above data, in a model of infection with Lm and endogenous T cell tracking, LP T cells, but not IEL, showed higher cytolitic potential than splenic T cells. Although these experiments were carried out at the peak of the effector (d9) and not at the memory phase, it is worth to notice that infection route seemed to affect the cytolytic potential of T cells in a compartment‐specific manner. In particular, LP T cells where more cytotoxic than splenocytes following oral infection, while splenic T cells lysed higher numbers of target cells following i.v. infection, hinting at a dependency of effector functions on infection route or anatomical location [9].

In humans, intestinal Trm were confirmed to produce cytokines and cytotoxic molecules. In particular, CD4^+^ T cells were found to produce high amounts of IFN‐γ, TNF‐α and IL‐2, among other cytokines; IL‐17 and GzmB were particularly prominent in the CD103^+^ fraction [89]. Of note, colonic and ileal Trm CD4^+^ produced the highest amount of IL‐17 among T cells recovered from multiple body locations [25]. Gut CD8^+^ Trm, were instead found to produce high levels of IFN‐γ, particularly in the small intestine [25].

CD8^+^ Trm cells in the mouse SI IEL showed low proliferation rates in comparison with splenic T cells, as assessed by BrdU incorporation [46], and such proliferation appeared to be IL‐15‐independent in the intestinal tissue, unlike in SLOs, kidney, and salivary glands [64]. CD4^+^ and CD8^+^ T cells in the human SI LP were similarly found to proliferate at very low rates at steady state [48, 90]. However, proliferation could be induced following cognate‐antigen stimulation in both intestinal CD4^+^ [13] and CD8^+^ T cells [53, 54], showing that these cells maintain the capacity to expand.

## Role of intestinal Trm in protection from infectious agents

The protective potential of intestinal Trm has been suggested by several studies, although the evidence appears less robust than for T cells residing at other anatomical locations, such as the skin, possibly due to the inherent difficulties in isolating the relative impact of these cells from the contribution of circulating cells [43].

Sheridan and collaborators showed that, following oral immunization with Lm, continuous treatment with an anti‐α4β7 antibody, which partly prevents migration of CD8^+^ T cells to the intestinal mucosa, resulted in reduced protection with the same pathogen from rechallenge thirty days later, with somewhat increased bacterial burdens in liver and MLNs [22].

We showed that an improved Lm‐OVA mucosal immunization approach, resulting in increased CD8^+^ Trm accumulation in the intestinal tissue but not in the spleen, enhanced protection from p.o. rechallenge with the pathogen, with a particularly significant effect in the intestinal tissue, but also in MLNs and spleen [24]. These data suggest that gut Trm play a crucial role in local protection from pathogen invasion. Of note, individual antibody‐mediated depletion of either CD8^+^, CD4^+^, or γδ T cells at the time of rechallenge did not impair protection, but simultaneous depletion of these three cell types did [24]; this is in agreement with a previous publication indicating that orogastric Lm infection promotes generation of multiple subsets of T cells, each contributing to protection [91].

Recent elegant fate mapping work employing p.o. Lm‐OVA infection, has uncovered key properties of intestinal Trm [53, 54]. These studies have convincingly proven that upon reinfection, not only do Trm proliferate extensively in situ expanding the local T cell pool, but they also generate a novel circulating effector cell progeny that contributes to systemic immune responses. Interestingly, part of these cells seeded draining‐lymph node generating LN‐resident T cells. The Tem cells generated from Trm, were transcriptionally similar, but not identical, to those originated from naïve or Tcm precursors, and displayed enhanced protective potential upon transfer, at least in the MLNs [53, 54].

One important implication of these studies is that experimental models aimed at separating the impact of circulatory T cells from that of Trm, might exclude important contributions from the Trm that regain circulating and effector capacities, such as ex‐Trm Tem.

## Role of intestinal Trm in protection from tumors

The role of Trm in protection from tumors has only begun to be elucidated. Skin resident Trm were shown to effectively limit the expansion of orthotopic melanoma in mouse models [92]. Similarly, using elegant parabiosis experiments in a mouse model of sub‐cutaneous melanoma grafting, Enamorado and collaborators showed that Trm provide additional protection to that conferred by circulating memory T cells [93].

Experiments carried out in models of orthotopic head and neck or lung cancers revealed that cancer vaccines delivered by the intranasal, but not intramuscular route, were highly effective. Such effectiveness depended on the expression of the mucosal integrin CD49a on CD8^+^ T cells, which was imprinted by mucosal, but not by splenic dendritic cells [94].

Strong evidence for an implication of intestinal Trm in anti‐cancer responses is to date missing. Honda and collaborators showed that improved systemic and colonic CD8^+^ T cell responses, obtained via provision of an *ad hoc* consortium of commensal microbes, enhanced tumor containment in a model of subcutaneous implantation of a colorectal cancer cell line [71]. Although in these experiments the relative contribution of circulating memory T cells was not dissected, it is worth noting that Trm can generate effector memory progenies with enhanced protective capacity, as mentioned above [55].

Indirect evidence suggests that in humans, Trm may have an important role in protecting against colorectal cancer (CRC). Infiltration of CD45RO^+^CD3^+^ T cells (as well as CD8^+^ T cells and GzmB^+^ cells) into CRC lesions correlates with highly improved prognosis, suggesting that T cells have strong therapeutic potential for the treatment of this tumor [95]. Although this early work did not investigate the expression of tissue residence markers in the infiltrating T cells, later studies found that CRC patients carrying DNA mismatch repair (MMR)‐deficient tumors display an intra‐tumoral increase of CD8^+^ T cells with a bona fide Trm phenotype (CD69^+^CD103^+^, also expressing PD1, FAS, HLA‐DR, CD38 and TIM3) [96]. Importantly, patients with MMR CRC were shown to respond significantly better to anti‐PD1 checkpoint blockade, raising the possibility that the therapy may act through intestinal Trm [97].

## Intestinal Trm in humans

Insights on intestinal Trm have also come from human studies, which have mostly encompassed flow cytometric and RNA‐seq analyses of biopsies from living donors as well as deceased individuals.

Seminal work from the laboratory of Donna Farber has defined the phenotype, transcriptional landscape, and functional properties of human Trm cells across organs, including the intestine [25, 44, 90]. By sampling organs from brain‐dead but otherwise healthy donors, which were explanted for transplantation, these studies confirmed that the near totality of CD4^+^ and CD8^+^ T cells found in the human intestine display an "effector memory" phenotype (CD45RA^−^, CD45RO^+^, largely CCR7^−^), while Trm in other organs comprise a substantial fraction of CCR7^+^ "central memory" T cells [25]. Intestinal Trms were found to proliferate at very low rates as compared to blood and LN T cells [90]. Furthermore, these cells expressed high levels of CD69 and CD103. IL‐17 production was restricted to intestinal T cells while IL‐2 and IFN‐γ were detected in Trm across all tissues.

Phenotyping based on CD28 and CD127 expression detected differences across organs and even within the intestinal tract, with CD4^+^ T cells expressing high levels of both markers in ileum and colon, and CD8^+^ displaying mostly a CD127 single‐positive phenotype in ileum and a double‐negative phenotype in colon. This was considered as a possible readout for signals received via IL‐7 (CD127 down‐regulation) or TCR stimulation (CD28 down‐regulation) at such different sites [90]. Notably, TCR signaling was found to be dispensable for Trm overtime maintenance in the gut, as well as in other organs, in a mouse model [88].

Contrary to what initially predicted, CD69 expression may not be associated with recent TCR activation in gut‐resident T cells, as indicated by comparative transcriptional analysis of CD69^+^ intestinal Trm and CD69^−^ splenic T cells [44]. Also, within organs, CD69^+^ cells showed distinct transcriptional profile from CD69^−^ cells. These studies identified a core transcriptional profile constituted by a set of genes being selectively up‐ or down‐regulated in mucosal Trm as compared to splenic cells. A comparison with a previously published mouse dataset identified large inter‐species differences, most notably the fact that human Trm from lungs did not express high levels of Hobit, which is instead a key master regulator for tissue residency, including the gut, in mice [44). This difference seems to be confirmed by the finding that, unlike in mice, human circulating T cells, and particularly effector CD8^+^ and cytotoxic CD4^+^, do express Hobit, which is crucial for their effector functions [98, 99]. However, despite such differences, Trm core genes identified in human T cells were significantly conserved in mouse Trm, including intestinal Trm. Analysis of transcriptional profiles across tissues, including the intestine, showed that human CD8^+^ Trm have relatively similar profiles across organs, while CD4^+^ Trm appear to be more variable [44].

By analyzing biopsies from sex‐ or HLA‐mismatched duodenal‐pancreatic transplant patients, Bartolome ´‐Casado et al. confirmed that tissue‐residence markers CD103 and CD69 were expressed in virtually all IEL and in the overwhelming majority of cells of donor origin (i.e. bona fide tissue‐resident) within the whole tissue [48]. Further, these cells presented a memory phenotype similar to that described in their murine counterparts, being largely CD45RO^+^, CD45RA^−^, CD62L^−^, CCR7^−^, and KLRG1^−^. Intestinal Trm proliferated at very low rates as indicated by Ki‐67 staining but were maintained at high numbers one year after transplant, with substantial replacement from cells of host origin occurring only for the minority of CD103^−^ cells in the lamina propria. CD103^−^ cells displayed phenotypic differences as compared to CD103^+^ LP and IEL CD8^+^ T cells, including a lower expression of CD127, and higher levels of PD‐1, NKG2D, KLRG1, and CD28 [48]. Notably, distinct phenotypic features and increased protective functions have been suggested for CD103^−^ LP cells also in animal models [62]. CD103^−^ T cells were replaced at a faster rate by recipient cells, suggesting that they may give rise to CD103^+^ cells once in the tissue. TCR sequencing of T cells isolated from two transplant patients detected some overlap between the repertoire at baseline (i.e. time of transplant) and 1 year post engraftment, with several expanded clonotypes being conserved and accounting for about 20% of the recovered population; 80% of T cell clones were instead identified as unique, however this number is probably an overestimation due to the limited sampling. Furthermore, and contrary to what suggested by previous mouse experiments [85], a strong correlation was found between the TCR repertoire in LP and IEL within each patient, suggesting productive cross‐talk, or shared clonal origin, for T cells in these two compartments. CD103^−^ cells were found to produce the highest amount of granzymes and perforin both at steady state and following stimulation, whereas CD103^+^ LP T cells produced the most cytokines both in terms of quantity and variety (polyfunctionality). IELs produced little of any of the above molecules in standard in vitro assays, suggesting that distinct signals may be necessary to activate them [85].

More recently, scRNA‐seq analyses in human intestinal Trm from transplant patients have uncovered the existence of two transcriptionally distinct subpopulations of CD69^+^ Trm, one expressing CD103^+^, which is in fact cytokine‐polyfunctional and expresses high levels of CD127, CCR6, and CD161, and one expressing β2 integrin (CD18), which is characterized by higher cytotoxic potential and expression of KLRG1 and MHC [34]. ITGAE^+^ cells also expressed specific transcription factors such as ZNF683 (Hobit) and JUN, while β2‐integrin^+^ expressed ZEB2, generally associated with terminally differentiated populations. Both populations expressed Runx3 and NR4A1. PRDM1 (the gene encoding for Blimp‐1) was highly expressed in CD4^+^ T cells, and less so in CD8^+^ T cells. Some residency‐associated genes were expressed differentially, such as ITGA1 in ITGAE^+^ and CRTAM in the β2‐integrin^+^. In this study, donor‐derived T cells were found in recipients up to five years post‐transplant [34]. Consistent with previous work, the authors confirmed that CD103 expression is restricted to CD69^+^ cells, and that a greater proportion of CD8^+^, as compared to CD4^+^ T cells, express CD103. Also in this report, recipient‐derived T cells had a substantially higher percentage of CD69^−^ cells, confirming that CD69 might be a good surrogate for residency at least in the intestinal tissue, although data from mouse experiment showed that this molecule is not required for the establishment of intestinal Trm [26]. Recipient‐derived Trm showed an over time increase in CD103 expression, indicating sequential acquisition of residency properties as shown also in the skin [31]. Proliferation of these T cells was confirmed to be rather low, and decreases from CD69^−^ to CD69^−^CD103^−^ cells. In early periods, infiltrating recipient cells have largely a CD69^−^ phenotype, but then they acquire the same phenotype as donor Trm and also cluster in those two subsets.

## Conclusions

The gastrointestinal tract is a major portal of entry for pathogens, and is situated in close proximity to the trillions of bacteria composing the gut microbiota. As a result, the intestinal tissue is heavily populated by resident memory T cells, which can readily reactivate in case of microbial invasion. These cells could potentially be harnessed by immunization strategies or through pharmacological interventions to enhance local pathogen or tumor clearance, or to prevent the onset of diseases.

Researchers have identified some of the crucial factors that regulate Trm generation and maintenance, highlighting that the unique environmental cues provided in different tissues, account for diverse specification signals and produce Trm with distinctive functional features. The advent of single‐cell analyses has expanded the depth to which such unique features can be detected. Upcoming work unraveling the pathways driving Trm generation and maintenance in the intestine, may lead to the design of novel therapeutic approaches to harness these cells against pathogens and tumors. In this context, it will be important to perform comparative evaluations of such signals between mouse models and human tissues, and to consider the specific local environment characterizing the different segments and layers of the GI tract, as wells as the factors that potentially modulate or perturb such distinct anatomical niches.

## Conflict of interest

The authors declare no commercial or financial conflict of interest.

**Abbreviations**
IEL: intra‐epithelial lymphocytes
LP: lamina propria
NLT: non‐lymphoid tissue
SI: small intestine
Trm: tissue‐resident memory T cells

## Data Availability

Data sharing is not applicable to this article as no new data were created or analyzed in this study.
